# A Predictive Risk Model for A(H7N9) Human Infections Based on Spatial-Temporal Autocorrelation and Risk Factors: China, 2013–2014

**DOI:** 10.3390/ijerph121214981

**Published:** 2015-12-01

**Authors:** Wen Dong, Kun Yang, Quan-Li Xu, Yu-Lian Yang

**Affiliations:** 1 School of Tourism and Geographic Science, Yunnan Normal University, Kunming 650500, China; dong_wen121@163.com (W.D.); go2happiness@163.com (Q.-L.X.); 2 School of Information Science and Technology, Yunnan Normal University, Kunming 650500, China; yulian_happy@163.com; 3 GIS Technology Engineering Research Centre for West-China Resources and Environment of Educational Ministry, Yunnan Normal University, Kunming 650500, China

**Keywords:** H7N9, avian influenza, spatial-temporal autocorrelation, risk factors, logistic regression modelling

## Abstract

This study investigated the spatial distribution, spatial autocorrelation, temporal cluster, spatial-temporal autocorrelation and probable risk factors of H7N9 outbreaks in humans from March 2013 to December 2014 in China. The results showed that the epidemic spread with significant spatial-temporal autocorrelation. In order to describe the spatial-temporal autocorrelation of H7N9, an improved model was developed by introducing a spatial-temporal factor in this paper. Logistic regression analyses were utilized to investigate the risk factors associated with their distribution, and nine risk factors were significantly associated with the occurrence of A(H7N9) human infections: the spatial-temporal factor φ (OR = 2546669.382, *p* < 0.001), migration route (OR = 0.993, *p* < 0.01), river (OR = 0.861, *p* < 0.001), lake(OR = 0.992, *p* < 0.001), road (OR = 0.906, *p* < 0.001), railway (OR = 0.980, *p* < 0.001), temperature (OR = 1.170, *p* < 0.01), precipitation (OR = 0.615, *p* < 0.001) and relative humidity (OR = 1.337, *p* < 0.001). The improved model obtained a better prediction performance and a higher fitting accuracy than the traditional model: in the improved model 90.1% (91/101) of the cases during February 2014 occurred in the high risk areas (the predictive risk > 0.70) of the predictive risk map, whereas 44.6% (45/101) of which overlaid on the high risk areas (the predictive risk > 0.70) for the traditional model, and the fitting accuracy of the improved model was 91.6% which was superior to the traditional model (86.1%). The predictive risk map generated based on the improved model revealed that the east and southeast of China were the high risk areas of A(H7N9) human infections in February 2014. These results provided baseline data for the control and prevention of future human infections.

## 1. Introduction

Following the human infections of H5N1, H9N2, H7N7, H7N2, H7N3 avian flu virus, in March 2013, the emergence of the novel avian-origin influenza A(H7N9) virus attracted worldwide concern about the possibility of a new influenza pandemic on human society [1,2,3]. A total of 460 A(H7N9) human infections with severe respiratory illness and mortality were confirmed from March 2013 to December 2014 in China [4]. The H7N9 epidemic essentially became a public health threat in China for almost two years. Gene sequence studies have demonstrated that the novel avian-origin influenza A(H7N9) virus combines with human receptors more easily than with birds, and virus may spread through the air [5]. Phylogenetic analysis of H7N9 showed that the virus was a novel triple reassortant of avian influenza A H7N3, A H7N9, and A H9N2 viruses [6,7,8]. Studies show that some areas of Eastern China have a higher risk of infection with non-uniform distribution [9].

The existing research results showed that closing live poultry market and suspension of live poultry trading were effective measures to contain the H7N9 outbreak, and this finding supported the possibility that live poultry markets and such environments contaminated by H7N9 virus were the most likely sources of human infection [7,10,11]. The closure of live poultry markets in China substantially reduced the incidence of human infection with novel avian-origin influenza A(H7N9) virus [12]. Few cases were reported in the summer of 2013 after some suitable public health measures such as closure of live poultry markets [13], but this was only temporary, and H7N9 infections in humans resurged in the winter of 2013 [10]. From January 2014 to April 2014 the outbreak of H7N9 peaked. In the summer of 2014 the epidemic reduced again, and by December 2014 the outbreak of H7N9 had relapsed once again. Although H7N9 family clusters have been reported in Shandong and Shanghai province on Xinhua News [14], so far there is no evidence of efficient or sustained human-to-human H7N9 virus transmission [15,16,17].

Logistic regression models are effective instruments to assess the infection risk of an epidemic. Currently there are two main types of logistic regression-based research in the field of human H7N9 virus infections: one is a comparison study, which aims to compare the differences between H7N9 outbreaks in humans or treatment effects among different people, and to find risk factors contributing to A(H7N9) human infections or treatment effects, thus probing for clues that are worth further study on the etiology and effective treatment of H7N9 [18,19,20,21,22,23,24]; the other is probability prediction, which analyses the relations between environmental factors and outbreaks in different environments, and establish a logistic regression model to estimate the probability of H7N9 outbreaks in humans and predict the possible trends of the epidemic in the future [25]. The latter research is relatively less common at present. A hypothesis of the traditional logistic regression model is that data must come from a random sample and all observations are independent of each other, so if there is a spatial or temporal autocorrelation between observations, the traditional logistic regression method will lead a deviation of the regression coefficient, and the probability prediction result is doubtful in this case. This paper studied the spatio-temporal characteristics of H7N9 outbreaks in humans from 2013 to 2014 and found that the distribution of the outbreak has significant spatial-temporal autocorrelation, so spatial-temporal autocorrelation must be considered in any predictive risk model of A(H7N9) human infections.

Spatial-temporal autocorrelation is a measurement of time and spatial correlation on the foundation of spatial autocorrelation with the further consideration of the time factor [26]. At present, research on reliable models including spatial-temporal autocorrelation for predicting the risk of A(H7N9) influenza is limited. Although some environmental factors (e.g., surface water availability, topography, climate, the distance to roads, poultry density, or human population density) have been associated with H7N9 outbreaks in humans in China, no analysis of spatial-temporal autocorrelation was made in that paper [25]. We believe that spatial-temporal autocorrelation can provide clues for understanding the spread mechanisms of the outbreak patterns of H7N9, and a more comprehensive study incorporating the analysis of the spatial-temporal factor and environmental factors is required in order to obtain more accurate information concerning the ecological risk of experiencing H7N9 in China. We included a spatial-temporal factor as an additional variable along with some traditional environmental factors such as mean monthly precipitation, mean monthly relative humidity, mean monthly temperature, roads and railways, migration routes, rivers and lakes, wetlands, and normalized difference vegetation index (NDVI). Finally, the experiments with the improved spatial-temporal autocorrelation model between the traditional logistic model showed the amelioration of the new model, and the new model was a useful complement and development of the traditional model.

H7N9 virus can infect birds without symptoms and it is difficult to monitor live poultry markets in a timely way, so sooner or later influenza A(H7N9) cases will appear again in the future, and assessing and predicting the risk of H7N9 pandemic are essential for controlling H7N9 outbreaks. Our objective in this study is tantamount to investigating the association between infected cases and risk factors, and this will provide useful information for developing effective counter measures aiming to interrupt the transmission cycle of H7N9 outbreaks. As a result, we have developed an improved risk prediction model by incorporating the spatial-temporal factor and environmental factors that would accurately and objectively evaluate the risk of A(H7N9) human infections in China, and the risk prediction can help the government to establish monitoring and preventive measures during the H7N9 epidemic.

## 2. Materials and Methods

### 2.1. Data Collection and Processing

This study focused on 460 human influenza A(H7N9) cases that occurred during 2013 and 2014 in China. All 460 of the cases were used to establish the predictive model, and the model was verified using the 101 cases that occurred in February 2014 because that month was a peak month of A(H7N9) human infections. Based on the cases and controls of A(H7N9) human infections, mean monthly precipitation, mean monthly relative humidity, mean monthly temperature, roads and railways, migration routes, rivers and lakes, wetlands, and NDVI, the GIS database of China was built.

We obtained information about the confirmed influenza A(H7N9) human cases from the World Health Organization (WHO) website [4]. Wetland data came from the State Forestry Administration of the People’s Republic of China website [27]. Vector data of the administrative divisions, national main roads and railways, river and lake were available from the National Fundamental Geographic Information System (China) [28]. NDVI data used in the study were derived from the Geospatial Data Cloud website [29]. Climate data of mean monthly precipitation, mean monthly relative humidity, mean monthly temperature were obtained from the Data Sharing Infrastructure of Earth System Science (China) [30]. The vector treatment of migratory routes was manufactured based on existing research results at present [31,32,33,34]. The population data of each affected area for 2013 and 2014 were obtained from the National Bureau of Statistics of the People’s Republic of China website [35].

### 2.2. Spatial Autocorrelation Analysis

Global Moran’s I statistic is usually used in spatial autocorrelation analysis [36]. In this paper Global Moran’s I analysis was carried out in ArcGIS 10.1 (ESRI Inc., Redlands, CA, USA) to examine the spatial distribution pattern of A(H7N9) human infections in China, which is given as [37]:

(1) $$I=\frac{n\sum_{i=1}^{n}\sum_{j=1}^{n}\omega_{i,j}(x_{i}-x)(x_{j}-x)}{(\sum_{i=1}^{n}\sum_{j=1}^{n}\omega_{i,j})\sum_{i=1}^{n}{\left(x_{i}-x\right)}^{2}},i\neq j$$

where *n* is equal to the total number of administrative regions in China, and ω*_i,j_* is the spatial weight between administrative region *i* and *j* which is automatically generated by the inverse distance method in this study, χ*_i_* is the number of influenza A(H7N9) cases in region *i*, and χ is the mean number of influenza A(H7N9) cases of all regions. The Global Moran’s index is bounded by −1.0 and 1.0.

The Z Score for the statistic is computed as [37]:

(2) $$Z=\frac{I-E[I]}{\sqrt{V[I]}}$$

where:

(3) $$E[I]=\frac{-1}{\left(n-1\right)}$$

(4) $$V[I]=E[I^{2}]-E{\left[I\right]}^{2}$$

If the *p*-value is not statistically significant, then we cannot reject the null hypothesis. It is quite possible that the spatial distribution of the cases is the result of random spatial processes. If the *p*-value is statistically significant, and the Z score is positive, the spatial distribution of the cases in the dataset is tending towards being spatially clustered. If the p-value is statistically significant, and the Z score is negative, the spatial distribution of the cases is tending towards being spatially dispersed.

### 2.3. Temporal Cluster Analysis

In this study, retrospective purely temporal analysis scanning employing a Discrete Poisson model was used to detect and evaluate the high risk temporal clusters of influenza A(H7N9) cases in 2013 and 2014 (SaTScan software, version 9.1.1) [38]. The scan statistic involves a flexible scanning window which gradually moves across time and scans for periods with high risk temporal clusters of the epidemic. During each time period, the number of observed and expected cases inside the window is compared to outside the window, and the greatest excess of observed cases are noted [39]. In the analysis process, the time aggregation length was set to one month, and the maximum temporal cluster size was set to 50% of study period to find possible clusters. Ensuring that the temporal cluster analysis has sufficient statistical power, the number of Monte Carlo replications was set to 999. The *p* values were obtained through Monte Carlo simulation and likelihood ratio tests were performed to determine the significance of identified clusters. If the *p* value was <0.05, the null hypothesis of a temporally random distribution was rejected [38,40].

### 2.4. Spatial-Temporal Factor Based on Descriptive Spatial-Temporal Statistics

#### 2.4.1. Descriptive Spatial-Temporal Statistics

**Figure 1:**
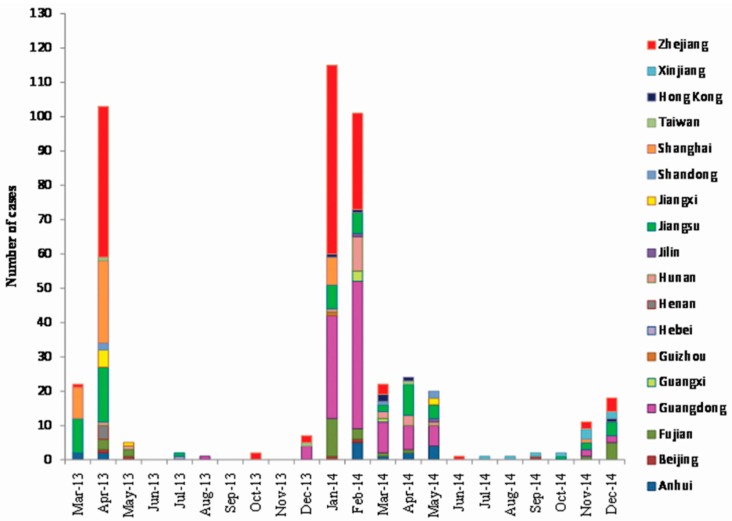
Monthly reported human cases of influenza A(H7N9) virus infection in China, March 2013–December 2014 (*n* = 460).

Figure 1 shows the monthly statistics of human cases of influenza A(H7N9) virus infection during 2013 and 2014 in China, which also includes the number of cases in each infected region. From the time perspective, cases were reported all year round, but in the spring and winter they occurred more often than at other times, and the two peaks of the disease appeared from March to April in 2013 (125 cases) and from January to May in 2014 (282 cases), respectively.

Besides apparent temporal clustering, the outbreak also displayed obvious regional clustering during the epidemic, as quite a few cases occurred in Zhejiang, Shanghai, Jiangsu, Guangdong and Fujian during the two epidemic peaks.

#### 2.4.2. Spatial Factor, Temporal Factor and Spatial-Temporal Factor

From the descriptive spatial-temporal statistics of the number of human cases of influenza A(H7N9) virus infection in 2013 and 2014 above, we can get some insights into the epidemic: there might be a certain spatial-temporal autocorrelation of H7N9 outbreaks because of the obvious regional clustering and temporal clustering of A(H7N9) human infections, which were linked to the time interval and the distance interval of the cases ocurred before. Intuitively, the smaller the time interval and the distance interval were, the greater the spatial-temporal autocorrelation was. Based on this, we put forward an argument that within a certain period of time a nearer distance between the sample and the historical case, or in a certain geographic range a shorter time interval between the sample and the historical case, all these might lead to a higher risk of A(H7N9) human infections in the location of the sample.

According to the analysis above, we defined the spatial factor, the temporal factor and the spatial-temporal factor of the sample point, and gave the methods of computing them as follows:

(I) We defined the spatial factor of the sample point δ as:

(5) $$\delta =\{\begin{array}{c} \begin{array}{cc} \frac{1}{1+s} & \text{(There is a history of A (H7N9) human infections within one month with the sample point.)} \end{array}\\ \begin{array}{ccc} 0 & & \text{(There is no history of A (H7N9) human infections within one month with the sample point.)} \end{array} \end{array}$$

where *s* is the shortest distance between the sample point and the historical cases points appeared within one month, when s equals 0, the sample point is in the same location with the historical case point and the weight reaches a maximum of 1. If there is no history of A(H7N9) human infections within one month with the sample point, the spatial factor δ is 0.

And (II) We defined the temporal factor of the sample point ρ*_i,j_* as:

(6) $$\rho_{i,j}=\rho_{j,i}=\{\begin{array}{c} \begin{array}{cc} \begin{array}{cc} \frac{1}{1+\left|t_{i}-t_{j}\right|} & \text{(There is a history of A (H7N9) human infections in the same city with the sample point.)} \end{array} & \end{array}\\ \begin{array}{ccc} 0 & & (\text{There is no history of A (H7N9) human infections in the same city with the sample point.)?} \end{array} \end{array}$$

where *t_j_* is the current time of the sample point and *t_i_* is the occurrence time of the recent human case which is in the same city as the sample point, and | *t_i_* − *t_j_* | is the time interval measured in days. If there is no history of A(H7N9) human infections in the same city as the sample point, the temporal factor ρ*_i,j_* is 0.

Finally (III) Based on the above two factors, and considering the spatial-temporal autocorrelation of A(H7N9) human infections, we finally defined the spatial-temporal factor of the sample point φ as:

(7) $$\varphi =\rho_{i,j}+\delta$$

where φ is the spatial-temporal factor of the sample point at a spatial location in the time of *t_j_*, and ρ*_i,j_* is the temporal factor, δ is the spatial factor of that sample point, respectively.

This spatial-temporal factor was introduced into the model as one of the risk factors to analyze its effect on A(H7N9) human infections in the subsequent modeling process.

### 2.5. Spatio-Temporal Sampling and Extracting Risk Factors

Four hundred and sixty H7N9 presence points representing case samples and 917 absence points representing control samples were generated throughout the country by spatio-temporal sampling rules defined as follows: (I) 460 influenza A(H7N9) cases occurred between March 2013 and December 2014 in China were collected as H7N9 case samples. (II) 917 control samples were uniformly generated in spatial terms by dividing a map of China (1:4,000,000 scale) into different regions according to the rule of a regular grid with 100 km × 100 km, and every centroid of the grid which didn’t overlap with the case samples was a control sample. Methods 1:2 matched case-control study was conducted. Finally (III) the time properties of case samples were the onset dates of human cases, and the time properties of the control samples were randomized between March 2013 and December 2014. In order to avoid biased sampling, and considering that the first peak of the epidemic appeared from March to April in 2013 (125 cases) which accounted for 27.2% of the total cases, and the second peak of the epidemic appeared from January to May in 2014 (282 cases) which accounted for 61.3% of the total cases, we followed an approach that 27.2% of the control samples’ time properties were randomized from March to April in 2013, 61.3% of which were randomized from January to May in 2014, and 11.5% of which were randomized during the remaining period of the H7N9 outbreak.

When extracting the values of risk factors, the sample points were overlaid on the map layers of environmental factors in the corresponding time. The values of mean monthly temperature, NDVI, mean monthly precipitation, and mean monthly relative humidity can be directly extracted by GIS, and other types of environmental factors such as roads, railways, migration routes, body of water and wetlands were obtained by calculating the nearest distance to the sample points in GIS. The spatial-temporal factors of the sample points were calculated using spatial-temporal factor function presented in this paper based on GIS spatial analysis.

### 2.6. Logistic Regression Analysis and Modelling

#### 2.6.1. Logistic Regression Analysis

Logistic regression analysis was utilized to examine the relationship between risk factors and the occurrence of A(H7N9) human infections. In this paper, based on the traditional logistic regression model [41], we present an improved model by introducing the spatial-temporal factor, and the improved model is defined as:

(8) $$P=\frac{\exp(\beta_{0}+\beta_{1}x_{1}+\ldots +\beta_{p}x_{p}+\gamma \varphi )}{1+\exp(\beta_{0}+\beta_{1}x_{1}+\ldots +\beta_{p}x_{p}+\gamma \varphi )}$$

where *P* is the probability of the occurrence of A(H7N9) human infection expressed as a number between 0 and 1, β_0_ is the constant, χ_1_,…, χ*_p_* are the key risk factors of the occurrence of A(H7N9) cases, and β_1_,…, β*_p_* are their regression coefficients, and specifically, φ is the spatial-temporal factor of a position in a certain space-time, and γ is its regression coefficient.

During this analysis, all laboratory tests were analyzed using univariate logistic regression and multivariate logistic regression in SPSS 19.0 (SPSS Inc., Chicago, IL, USA). Firstly, risk factors associated with A(H7N9) human infections were analyzed by univariate logistic regression, which was applied to investigate the effects of each risk factor on the occurrence of A(H7N9) cases in humans. Risk factors that were statistically significant at *p*-value < 0.05 were included for multivariable logistic regression analysis. Backward Wald method was applied to exclude the risk factors with *p*-value > 0.05. The classification cut off was 0.5 and the maximum number of iterations was 20.

The mean odds ratios (OR), 95% confidence intervals (CIs) of OR, and *p*-value were considered to be indicators of model performance both in univariate logistic regression and multivariate logistic regression. And there is a corresponding relationship between the regression coefficient (such as β_1_,…, β*_p_* , and γ in Equation(8)) and the mean odds ratios(OR) as:

(9) $$OR_{j}=\exp(\beta_{j})$$

where β*_j_* is the regression coefficient of the risk factor χ*_j_*, if β*_j_* = 0, then *OR_j_* = 1, this means the risk factor χ*_j_* has no effect on the occurrence of A(H7N9) cases in humans; if β*_j_* > 0, then *OR_j_* > 1, this means the risk factor χ*_j_* has a positive effect on the occurrence of cases; if β*_j_* < 0, then *OR_j_* < 1, it means the risk factor χ*_j_* has a negative effect on the occurrence of cases.

The *p*-value represents the value of the significance test, and only significant risk factors (*p*-value < 0.05) were retained in the model. When the regression coefficients are identified, we can use the model to calculate the possibility of H7N9 outbreaks in humans depending on the values of risk factors.

#### 2.6.2. Model Validation

Based on the traditional logistic regression model and the improved logistic regression model, two predictive risk maps of H7N9 outbreaks in humans for China were created using ArcGIS 10.1 (ESRI Inc., Redlands, CA, USA), and the 101 influenza A(H7N9) cases during February 2014 (a peak month) were used to compare and validate the predictive results of the two models. In this process, the spatial positions of predicting samples were the centroids of the regular grid, and time properties of predicting samples were randomized in February 2014. For the traditional logistic regression model, the risk probability of predicting samples was calculated by running the model only once because the risk factors in which were invariant within this month. But for the improved logistic regression model, we ran the model ten times and took the average risk as the risk probability of predicting samples because of the variable spatial-temporal factor.

## 3. Results

### 3.1. Case Analysis

**Figure 2:**
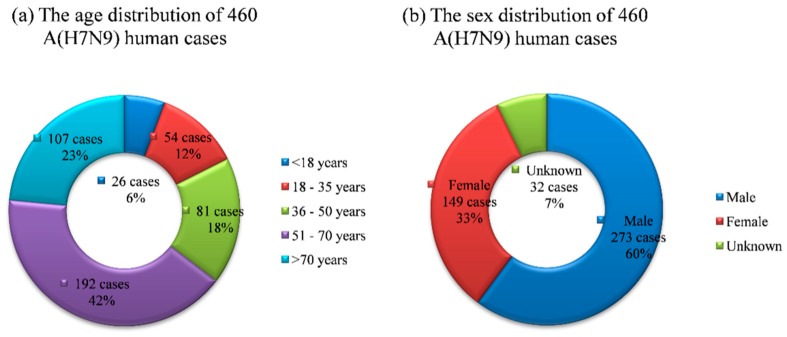
The age and sex distribution of 460 influenza A(H7N9) human cases reported in China during 2013 and 2014.

By analyzing the age and sex distribution of 460 influenza A(H7N9) human cases reported in China during 2013 and 2014 (Figure 2), we can get some useful information about the epidemic. The age range of cases which was from 0.5 to 91 years old was quite considerable, and the age distribution of cases showed about 6% under age 18, 12% between the ages of 18–35 years, 18% between 36 and 50 years, 42% between the ages of 51–70 and 23% above age 70, and the majority of H7N9-infected were over 50 years old. The male to female ratio of cases was 1.83:1 without considering the 32 cases lacking gender information, so males ran higher risk than females.

### 3.2. Spatial Distribution of A(H7N9) Human Infections

**Figure 3:**
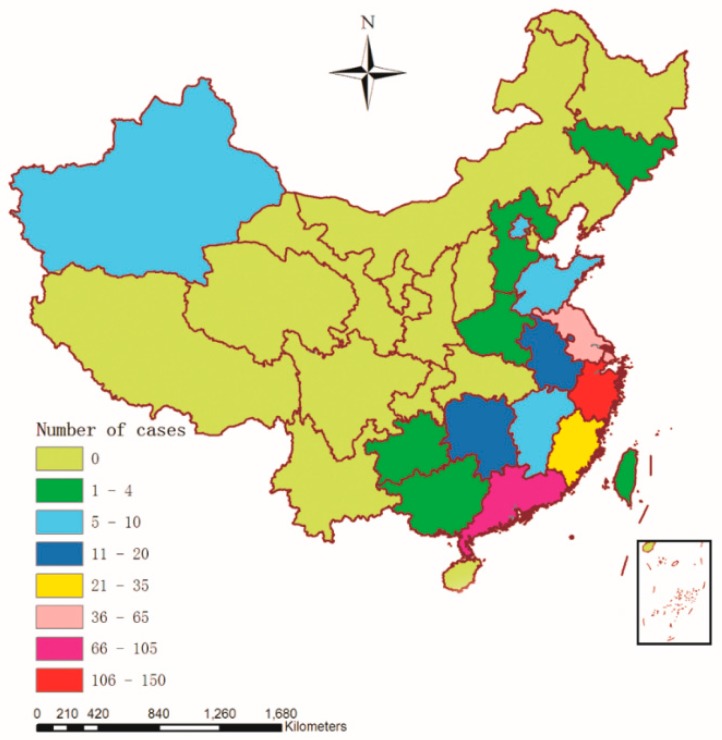
H7N9 persistence as measured by the cumulative number with reported outbreaks (March 2013 to December 2014) in China (*n* = 460).

Geographical analysis is a complementary instrument of public health surveillance that can analyze a disease’s spatial distribution within a territory [42]. By collecting and processing the data of influenza A(H7N9) human cases in GIS, a map displaying the spatial distribution of the affected provinces and municipalities was created with a digital topographic map of China (1:4,000,000 scale) as the background (Figure 3), which provided an overall impression about the spatial distribution of A(H7N9) human infections from March 2013 to December 2014 in China.

As we can see in Figure 3, there were 18 provinces and municipalities with A(H7N9) human infections between March 2013 and December 2014 in China, and the southeast coast of China was the high incidence area of A(H7N9) human infections, including Shanghai (42), Zhejiang (142), Guangdong (104), Jiangsu (62) and Fujian (27), which accounted for about 81.96% of influenza A(H7N9) cases (377/460). The remaining cases were scattered in Hunan (19), Anhui (16), Xinjiang (9), Jiangxi (8), Beijing (5), Shandong (5), Hong Kong (6), and so on.

### 3.3. Spatial Autocorrelation Analysis

The spatial autocorrelation analysis of the distribution of A(H7N9) human infections in 2013 and 2014 in China was analyzed by Global Moran’s I statistic in ArcGIS 10.1 (ESRI Inc., Redlands, CA, USA). The results showed that the Moran’s I index values were positive (Moran’s I = 0.256836 and 0.073536 in 2013 and 2014, respectively) and they were larger than the Expected index value (Expected index = −0.030303 both in 2013 and 2014), the *p*-values were 0.000032 and 0.019727 in 2013 and 2014, respectively (<0.05), and the Z Scores were all positive (Z = 4.158882 and 2.331500 in 2013 and 2014, respectively) (Table 1). Because the *p*-values were statistically significant and the Z Scores were positive, we could reject the null hypothesis, and the spatial distribution of A(H7N9) human infections is more spatially clustered than would be expected, so there was an obvious spatial autocorrelation in the distribution of A(H7N9) human infections both in 2013 and 2014.

**Table 1:** Result of the global autocorrelation analysis on distribution of A(H7N9) human infections’ number in 2013 and 2014 in China.

Year	Moran’ I	Expected Index	Variance	Z Score	*p*-Value	Result
2013	0.256836	−0.030303	0.004767	4.158882	0.000032	Clustered
2014	0.073536	−0.030303	0.001984	2.331500	0.019727	Clustered

### 3.4. Temporal Cluster Analysis

The results of the temporal cluster analysis of influenza A(H7N9) cases in 2013 and 2014 (China) indicated the seasonal tendency of H7N9 transmission, which showed the epidemic was not distributed randomly but significantly clustered on the time dimension (Table 2). Using the maximum temporal cluster size of 50% of study period, each year there was one most likely cluster identified for 2013 and 2014. The RR of the cluster detected in 2013 was 9.30 (*p* = 0.001) with an observed number of cases of 125 compared with an expected number of cases of 62.70 and the time frame was 1 March 2013 to 30 April 2013, and a lower RR of 2.24 was found in 2014 (*p* = 0.001, Obs = 282, Exp = 247.27) with the time frame of 1 January 2014 to 31 May 2014, which suggested there was a weaker temporal cluster in 2014. There was no secondary cluster identified in 2013 and 2014.

**Table 2:** The temporal clusters of influenza A(H7N9) cases detected using the temporal cluster analysis, China, 2013–2014.

Type	Year	Time Frame	Obs	Exp	LLR	RR	*p* Value
Most likely	2013	2013/3/1 to 2013/4/30	125	62.70	60.065300	9.30	0.001
Most likely	2014	2014/1/1 to 2014/5/31	282	247.27	12.751988	2.24	0.001
Secondary	-	-	-	-	-	-	-

Obs—the number of observed cases in a cluster; Exp—the number of expected cases in a cluster; LLR—Log likelihood ratio; RR—Relative risk.

### 3.5. Modelling and Analysis

From the results of the spatial autocorrelation analysis and the temporal cluster analysis of influenza A(H7N9) cases in 2013 and 2014 (China) above, we can prove that there was a certain spatial-temporal autocorrelation of A(H7N9) human infections, which is in consistent with the result from the descriptive spatial-temporal statistics of the H7N9 outbreak (see Methods section), so it is reasonable to consider and introduce the spatial-temporal factor in the traditional logistic regression model.

#### 3.5.1. Spatio-Temporal Sampling and Extracting Risk Factors

**Figure 4:**
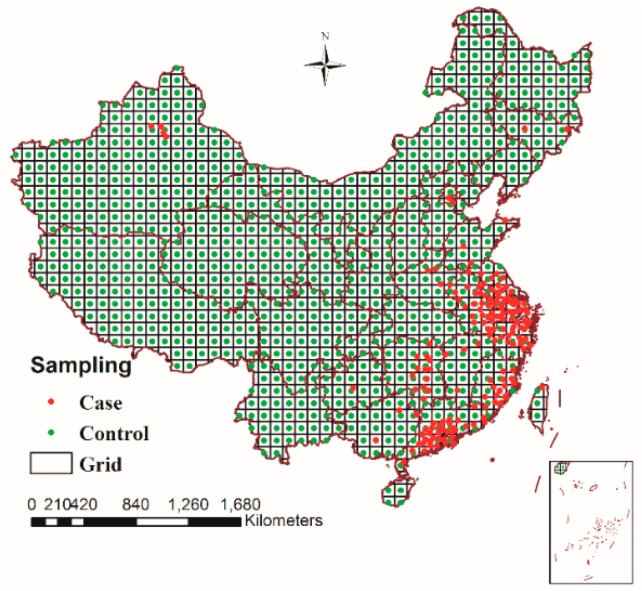
Sampling results of 460 H7N9 case samples (red points) and 917 control samples (green points), March 2013–December 2014, China.

According to the sampling rules defined earlier, the sampling results were shown in Figure 4: the black grid was the range of sampling which was generated by dividing a map of China (1:4,000,000 scale) into different regions with a 100 km × 100 km regular grid, where 460 H7N9 case samples (red points) occurred between March 2013 and December 2014 in China and 917 control samples (green points) with random time properties in the time range defined by the sampling rules were generated throughout the country. All of these case samples and control samples had definite localization in space and time properties, so we can extract the corresponding risk factors and calculate spatial-temporal factors for all of the samples.

**Figure 5:**
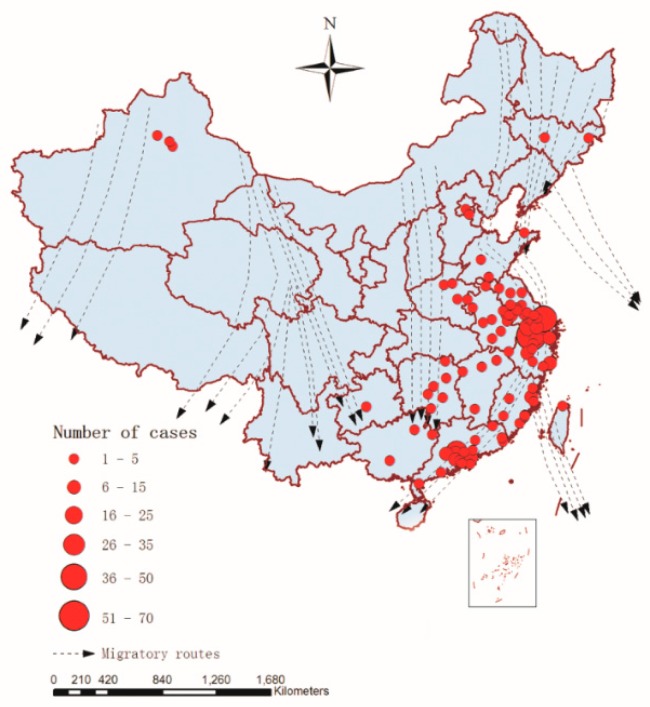
Migratory routes and spatial distribution of human cases of influenza A(H7N9) virus infection (*n* = 460) in 18 provinces and municipalities, China, March 2013–December 2014.

When extracting the values of the risk factors, all of them could be directly extracted and calculated by GIS, and the spatial-temporal factors of the sample points were calculated by spatial-temporal factor function presented in this study based on GIS spatial analysis. Among them, in order to calculate the nearest distance between migration routes and the sample point, the vector treatment of migratory routes was made on the base of existing research results at present [31,32,33,34], which was overlaid with the number of human cases of influenza A(H7N9) virus infection from March 2013 to December 2014 in China. The result was shown in Figure 5, which showed that regions with high incidence of H7N9 from March 2013 to December 2014 in China and eastern migratory routes were rather closely related, especially in the central and southern regions of the eastern migratory routes.

#### 3.5.2. Univariable Logistic Regression Analysis

Because different risk factors varied significantly, we conducted univariable analysis on the training samples (460 case samples and 917 control samples) to determine which risk factors were significant in the logistic regression model, and the significant risk factors entered into the multivariable logistic regression analysis. The risk factors used in this study are shown in Table 3.

All of the risk factors (including road and railway, river and lake, mean monthly temperature, wetland, migration route, spatial-temporal factor, relative humidity, mean monthly precipitation and NDVI) were evaluated by univariable logistic regression analysis in the SPSS software (version 19.0). Results of the univariable logistic analysis are shown in Table 4. The univariable analysis revealed that all of the eleven risk factors yielded *p*-values < 0.05 and were subsequently included in the multivariable analysis.

**Table 3:** Summary of risk factors used in the logistic regression analysis of human infection with avian influenza A(H7N9) virus in China, with abbreviation and unit.

Risk Factors	Abbreviation	Description of Factors	Unit
railway	rw	Distance to the nearest railway	km
migration route	mig	Distance to the nearest migration route	km
spatial-temporal factor	φ	Spatial-temporal factor	No unit
wetland	w	Minimal distance to the nearest wetland	km
temperature	tem	Mean monthly temperature	°C
NDVI	vi	Normalized differential vegetation index	No unit
river	r	Minimal distance to the nearest river	km
lake	l	Minimal distance to the nearest lake	km
road	ro	Distance to the nearest main road	km
relative humidity	rh	Mean monthly relative humidity	%
precipitation	prec	Mean monthly precipitation	cm

**Table 4:** Univariate analysis of risk factors for human infection with avian influenza A(H7N9) virus in China, March 2013–December 2014.

Risk Factors	B	OR (95% CI)	*p*-Value
r	−0.275	0.759 (0.708–0.815)	<0.001
l	−0.010	0.990 (0.987–0.993)	<0.001
ro	−0.264	0.768 (0.717–0.822)	<0.001
rw	−0.052	0.949 (0.935–0.964)	<0.001
w	−0.005	0.995 (0.993–0.996)	0.001
tem	0.103	1.109 (1.070–1.149)	<0.001
vi	1.519	4.568 (1.807–11.549)	0.003
rh	0.259	1.295 (1.197–1.402)	<0.001
prec	0.103	1.108 (1.029–1.193)	0.001
mig	−0.033	0.967 (0.962–0.973)	<0.001
φ	27.698	1.070E12 (3.070E9–3.728E14)	<0.001

B—regression coefficient of the factor; OR—odds ratios; 95% CI—95% confidence interval; *p*-value—the value of the significance test.

#### 3.5.3. Multivariable Logistic Regression Analysis

In the univariate analysis, all of the risk factors remained significant and were associated with a high risk of infection with influenza A(H7N9) (*p* < 0.05) (Table 4), so all of them were considered for inclusion in the multivariable logistic regression analysis. Based on the training samples (460 case samples and 917 control samples), we used two kinds of methods to conduct multivariable logistic regression analysis (traditional logistic regression analysis and improved logistic regression analysis), and compared the different results of these two methods. The results of multivariable logistic regression analysis using traditional logistic regression and improved logistic regression are presented in Table 5. The fitting accuracy of the two methods is shown in Table 6.

**Table 5:** Results of multivariate analysis of risk factors for human cases of influenza A(H7N9) virus infection using traditional logistic regression and improved logistic regression, March 2013–December 2014, China.

Risk Factors	Traditional Multivariate Analysis			Improved Multivariate Analysis		
	B	OR (95% CI)	*p*-Value	B	OR (95% CI)	*p*-Value
r	−0.138	0.872 (0.803–0.946)	<0.001	−0.150	0.861 (0.798–0.929)	<0.001
l	−0.009	0.991 (0.987–0.995)	0.001	−0.008	0.992 (0.989–0.996)	<0.001
ro	−0.080	0.923 (0.887–0.961)	<0.001	−0.099	0.906 (0.868–0.946)	<0.001
rw	−0.040	0.961 (0.933–0.989)	0.001	−0.020	0.980 (0.970–0.991)	<0.001
tem	0.109	1.116 (1.026–1.213)	<0.001	0.157	1.170 (1.099–1.245)	0.001
rh	0.274	1.316 (1.110–1.560)	<0.001	0.291	1.337 (1.211–1.477)	<0.001
prec	−0.401	0.669 (0.531–0.844)	<0.001	−0.487	0.615 (0.502–0.752)	<0.001
mig	−0.016	0.984 (0.978–0.990)	<0.001	−0.007	0.993 (0.990–0.997)	0.001
φ	-	-	-	14.750	2,546,669.382 (220,444.087–29,420,271.688)	<0.001
Constant	−20.838	0.000	<0.001	−20.096	0.000	<0.001

B—regression coefficient of the factor; OR—odds ratios; 95% CI—95% confidence interval; *p*-value—the value of the significance test; Wetland and NDVI did not enter the two final models (*p* > 0.05).

**Table 6:** The fitting accuracy of the traditional model and the improved model calculated by multivariable logistic regression analysis.

Observed	Predicted Results from Traditional Logistic Regression Model			Predicted Results from Improved Logistic Regression Model		
	0	1	Percentage Correct	0	1	Percentage Correct
0 (917)	828	89	90.3	851	66	92.8
1 (460)	102	358	77.8	50	410	89.1
Overall Percentage			86.1			91.6

1—the case sample; 0—the control sample; The cut value is 0.500.

In the traditional multivariate logistic regression model, river (OR = 0.872, *p* < 0.001), lake (OR = 0.991, *p* < 0.05), road (OR = 0.923, *p* < 0.001), railway (OR = 0.961, *p* < 0.05), migration route (OR = 0.984, *p* < 0.001), temperature (OR = 1.116, *p* < 0.001), precipitation (OR = 0.669, *p* < 0.001) and relative humidity (OR = 1.316, *p* < 0.001) were significantly associated with human cases of influenza A(H7N9) virus infection. Among them, temperature and relative humidity were the most significant risk factor for human influenza A(H7N9) cases in China (OR > 1). The difference of wetland and NDVI was not statistically significant (*p* > 0.05), so they didn’t enter the final traditional model. Through multi-step iteration, the fitting accuracy of the traditional logistic regression model was 86.1% (Table 6), so the risk factors involved in the model had great influence on the H7N9 epidemic.

An improved model was proposed by introducing the spatial-temporal factor φ in traditional logistic model, we included φ as an additional variable along with other risk factors used in traditional logistic regression analysis. In the improved multivariate logistic regression model, we noted that the difference of the spatial-temporal factor φ was obviously statistically significant, and φ was a very significant risk factor for human influenza A(H7N9) cases in China (OR = 2546669.382, *p* < 0.001), this showed the existence of spatial-temporal autocorrelation in A(H7N9) human infections. In addition, migration route (OR = 0.993, *p* < 0.01), river (OR = 0.861, *p* < 0.001), lake (OR = 0.992, *p* < 0.001), road (OR = 0.906, *p* < 0.001), railway (OR = 0.980, *p* < 0.001), temperature (OR = 1.170, *p* < 0.01), precipitation (OR = 0.615, *p* < 0.001) and relative humidity (OR = 1.337, *p* < 0.001) were also significantly associated with A(H7N9) human infections. Wetland and NDVI didn’t enter the final improved model too (*p* > 0.05). Through multi-step iteration, the fitting accuracy of the improved model was 91.6%, which was superior to traditional logistic regression analysis (86.1%) (Table 6). According to the regression coefficients obtained in Table 5, we can get two predictive risk models produced by the traditional logistic regression and improved logistic regression as follows:

(10) $$p=\frac{\text{exp(‒20.838‒0.138*}r\text{‒0.009*}l\text{‒0.08*}ro\text{‒0.04*}rw\text{+0.109*}temp\text{‒0.401*}prec\text{+0.274*}rh\text{‒0.016*}mig)}{1+\text{exp(‒20.838‒0.138*}r\text{‒0.009*}l\text{‒0.08*}ro\text{‒0.04*}rw\text{+0.109*}temp\text{‒0.401*}prec\text{+0.274*}rh\text{‒0.016*}mig)}$$

(11) $$p=\frac{\text{exp(‒20.096‒0.15*}r\text{‒0.008*}l\text{‒0.099*}ro\text{‒0.02*}rw\text{+0.157*}temp\text{‒0.487*}prec\text{+0.291*}rh\text{‒0.007*}mig\text{+14.750*}\varphi )}{1+\text{exp(‒20.096‒0.15*}r\text{‒0.008*}l\text{‒0.099*}ro\text{‒0.02*}rw\text{+0.157*}temp\text{‒0.487*}prec\text{+0.291*}rh\text{‒0.007*}mig\text{+14.750*}\varphi )}$$

Equation (10) is obtained by the traditional logistic regression, and Equation (11) is obtained by the improved logistic regression, respectively.

### 3.6. Prediction and Validation

**Figure 6:**
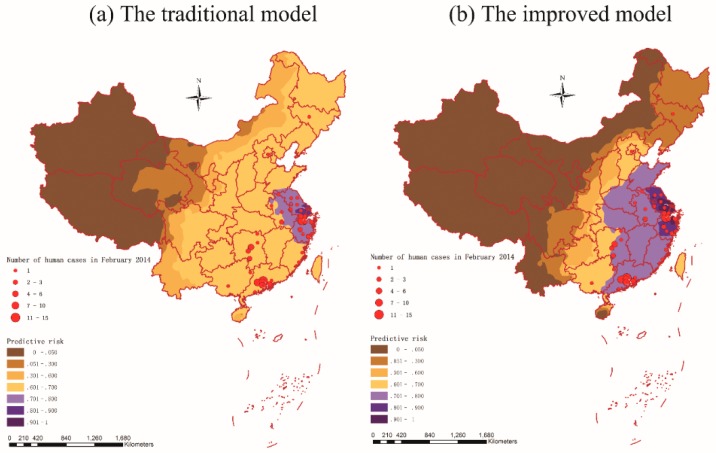
Predictive risk maps of A(H7N9) human infections in China based on the traditional logistic regression model (**a**) and the improved logistic regression model (**b**). Locations of the 101 influenza A(H7N9) cases in February 2014 are also indicated in the two maps.

Two predictive risk maps of H7N9 outbreaks in humans for China were generated and presented based on the two predictive risk models defined in Equations (10) and (11) above (Figure 6). In order to check the agreement of outbreak locations with predicted risk subdistricts by the two models, locations of the 101 A(H7N9) human cases during February 2014 (a peak month) in China were also plotted into the two predictive maps and overlay analysis was performed in ArcGIS 10.1.

Overlay analysis of locations of human cases during February 2014 on the two predicted probability maps got the results: for the predictive risk map generated by the traditional logistic regression model, 44.6% (45/101) of the cases overlaid on the high risk areas (the predictive risk > 0.70), but for the another one generated by the improved logistic regression model, there were 90.1% (91/101) of the cases occurred in the high risk areas (the predictive risk > 0.70). The validation results showed the improved model was more accurate and efficient compared with the traditional model, and the high risk areas of A(H7N9) human infections in February 2014 presented by the improved model were mainly located in the east and southeast of China.

As we can see in Figure 6, there were 10 provinces involving A(H7N9) human infections during February 2014 in China. Among them, Guangdong (43), Zhejiang (28) and Hunan (10) were the high incidence areas of A(H7N9) human infections, which accounted for about 80.2% of influenza A(H7N9) cases (81/101). There was an obvious regional difference in risk prediction between the two models, for the improved model, those three provinces were all located in the high risk areas (the predictive risk > 0.70), but for the traditional model, only Zhejiang was located in the high risk areas (the predictive risk > 0.70). Thus it can be seen that the improved model was more sensitive to H7N9 outbreaks than the traditional model. This may be because that the improved model not only included the environmental factors, but also took account the spatial-temporal factor which could depict the spatial-temporal autocorrelation of A(H7N9) human infections.

## 4. Discussion

The emergence of human infection with novel avian-origin influenza A(H7N9) virus in China has posed significant threats to both the public and the society. In this study, we investigated the spatial-temporal distribution features of A(H7N9) human infections in China from 2013 to 2014 and found that the distribution of the outbreak has significant spatial-temporal autocorrelation. Based on this we developed an improved logistic regression model by introducing a spatial-temporal factor, and identified the risk factors favoring the occurrences of H7N9 outbreaks and predicted the risk of A(H7N9) human infections by using the improved model.

Through the analysis of the spatial distribution characteristics of A(H7N9) human infections, our studies found that generally the southeast coast of China was the high incidence area of H7N9 outbreaks in humans. Especially, Shanghai, Zhejiang, Guangdong, Jiangsu and Fujian were observed an obvious regional clustering, this might because that the five areas are economically developed areas and connected with each other both in territory and economy, where the geomorphological environments and living environments are very close to each other, and such environments in this five areas may be suitable for the spreading of H7N9 virus. During the epidemic, most of the affected regions are middle- and large-sized cities which have a big population and developed economy. However, it is worth noting that there were nine human infection cases identified in Xinjiang from July 2014 to December 2014, away from the southeast coast of China and where no case had occurred before. This phenomenon is worth serious consideration in future research.

Some researchers have suggested that the spatial-temporal distribution of H7N9 in China between 2013 and 2014 presented different forms such as strong space-time clustering, weak space-time clustering or spatial-temporally randomly distributed in different areas and times [43], but from the results of our study we found that there was still an obvious spatial-temporal clustering during the epidemic as a whole. For example, by the spatial autocorrelation analysis we found that there was an apparent spatial autocorrelation totally in the distribution of influenza A(H7N9) cases, and by the temporal cluster analysis we identified two most likely clusters which had a significantly (*p* < 0.001) increased H7N9 risk with the time frame of 1 March 2013 to 30 April 2013 and 1 January 2014 to 31 May 2014. All of this suggested that the spatial-temporal distribution of H7N9 in China were not random but with a certain spatial-temporal autocorrelation overall. To solve this problem, we defined the spatial-temporal factor φ of A(H7N9) human infections which was introduced into the traditional logistic model as one of the risk factors, and found that the difference of the spatial-temporal factor φ was obviously statistically significant (*p* < 0.001). Therefore, it is reasonable to introduce the spatial-temporal factor in traditional logistic model.

During modeling, we identified nine risk factors for A(H7N9) human infections in the improved logistic regression model: spatial-temporal factor φ; mean monthly precipitation; mean monthly relative humidity; mean monthly temperature; distance to the nearest migration route, river, road, railway and lake (*p* < 0.01). We could draw some conclusions or suggestions from the relationship between risk factors and the occurrence of A(H7N9) cases in humans: (I): A significant positive effect was observed for spatial-temporal factor φ, which was a very significant risk factor for human influenza A(H7N9) cases in China (OR = 2546669.382, *p* < 0.001). This may be because that a nearer distance and a shorter time interval between the sample and the historical case will lead to a higher value of the spatial-temporal factor, thus raising the risk of H7N9 infections. (II): A(H7N9) human infections were strongly associated with an increased mean monthly relative humidity and lower mean monthly precipitation. Significant negative effects for mean monthly precipitation (OR = 0.615, *p* < 0.001) and significant positive effects for mean monthly relative humidity (OR = 1.337, *p* < 0.001) were observed in the final model, which indicated that reduced precipitation and increased relative humidity might increase the probability of A(H7N9) human infections. This is perhaps because of the sensitivity of H7N9 virus to climate, and the virus may survive longer in an environment like this. In fact, research has shown that H7N9 incidence rate was significantly associated with mean rainfall [44], which was consistent with our results for precipitation. (III): There was a significant negative association between the distance to the nearest river (OR = 0.861, *p* < 0.001), lake (OR = 0.992, *p* < 0.001) and A(H7N9) human infections, so the distance to the nearest river and lake was also crucial factors which affected H7N9 outbreaks in humans. The closer distance from lakes and rivers, the greater the risk of A(H7N9) human infections. This might because the water bodies have a higher exposure risk than other environmental conditions as an important habitat for birds. (IV): There was a positive association between A(H7N9) human infections and mean monthly temperature (OR = 1.170, *p* < 0.01), so the regions with higher temperature were likely to appear at higher risk of H7N9 epidemic within a certain range of temperatures. A previous study has highlighted that optimal temperature (7 °C to 15 °C) may be one of the driving forces for H7N9 [44]. Therefore A(H7N9) human infections were significantly associated with mean monthly temperature. (V): In the final model, we found a significant negative association between the distance to the nearest main road (OR = 0.906, *p* < 0.001), railway (OR = 0.980, *p* < 0.001) and A(H7N9) human infections. As an important traffic facility across the country, transportation increased the potential and opportunities for spatial disease spread, which may be a risk factor contributing to A(H7N9) human infections. And (VI): There was a negative association between the distance to the nearest migration route (OR = 0.993, *p* < 0.01), this was probably because avian influenza viruses could be introduced into poultry through migratory wild birds [45], and the outbreak might have a certain affinity with migratory routes in spatial terms. (VII): Although some A(H7N9) human infections presented gathered around the wetlands, wetlands did not enter the final multivariable logistic regression analysis model. This might because the climatic conditions (temperature, humidity, precipitation, *etc.*) were not conducive to the survival and reproduction of H7N9 virus, which reduced the occurrence of the epidemic. As an important part of the environment, vegetation can provide a good living environment for birds, but NDVI did not get into the final model either. This might be a consequence of interaction with other risk factors in the multivariable logistic regression analysis.

## 5. Conclusions

In conclusion, this study examined the epidemiologic characteristics of A(H7N9) human infections in China, such as the spatial and temporal distribution, spatial-temporal autocorrelation of H7N9, probable risk factors, and so on. The results showed that the epidemic spread with significant spatial-temporal autocorrelation. However, the traditional logistic regression model cannot depict the spatial-temporal autocorrelation of A(H7N9) human infections, so in this paper an improved model including nine risk factors was developed by introducing a spatial-temporal factor in the traditional logistic regression model. Through comparison with the traditional model without considering spatial-temporal factor, the improved model showed a higher accuracy of fitting and a better risk prediction effect. Besides, the spatial-temporal factor φ was statistically significant in the multivariable logistic regression analysis, which indicated that the spatial-temporal factor has an important effect on the occurrence of A(H7N9) human infections. The predictive risk map generated by the improved model could be used for identifying priority areas to implement preventive intervention so as to reduce the risk of future infections.

Because the spread of A(H7N9) human infections is quite a complex process, some factors are difficult to quantify in the model (such as government policy), and this has influenced the accuracy of the model to a certain extent. Further studies are also required to delineate the mechanisms of A(H7N9) human infection transmission. Above all, knowledge of risk factors discussed in our paper should help fight potential A(H7N9) human infections, and the risk prediction by the final model could help the government to establish monitoring and preventive measures during future H7N9 epidemics.
